# Superhydrophobic surfaces of the water bug *Notonecta glauca*: a model for friction reduction and air retention

**DOI:** 10.3762/bjnano.2.17

**Published:** 2011-03-10

**Authors:** Petra Ditsche-Kuru, Erik S Schneider, Jan-Erik Melskotte, Martin Brede, Alfred Leder, Wilhelm Barthlott

**Affiliations:** 1 Nees Institute for Biodiversity of Plants, Rheinische Friedrich-Wilhelms University of Bonn, Meckenheimer Allee 170, Bonn, 53115, Germany; 2 Department of Zoology: Functional Morphology and Biomechanics, Christian-Albrechts-University of Kiel, Am Botanischen Garten 1–9, Kiel, 24098, Germany; 3 Lehrstuhl Strömungsmechanik, Universität Rostock, Albert Einstein Str. 2, Rostock, 18051, Germany

**Keywords:** air film, aquatic insects, backswimmer, drag reduction, superhydrophobic surfaces

## Abstract

Superhydrophobic surfaces of plants and animals are of great interest for biomimetic applications. Whereas the self-cleaning properties of superhydrophobic surfaces have been extensively investigated, their ability to retain an air film while submerged under water has not, in the past, received much attention. Nevertheless, air retaining surfaces are of great economic and ecological interest because an air film can reduce friction of solid bodies sliding through the water. This opens perspectives for biomimetic applications such as low friction fluid transport or friction reduction on ship hulls. For such applications the durability of the air film is most important. While the air film on most superhydrophobic surfaces usually lasts no longer than a few days, a few semi-aquatic plants and insects are able to hold an air film over a longer time period. Currently, we found high air film persistence under hydrostatic conditions for the elytra of the backswimmer *Notonecta glauca* which we therefore have chosen for further investigations. In this study, we compare the micro- and nanostructure of selected body parts (sternites, upper side of elytra, underside of elytra) in reference to their air retaining properties. Our investigations demonstrate outstanding air film persistence of the upper side of the elytra of *Notonecta glauca* under hydrostatic and hydrodynamic conditions. This hierarchically structured surface was able to hold a complete air film under hydrostatic conditions for longer than 130 days while on other body parts with simple structures the air film showed gaps (underside of elytra) or even vanished completely after a few days (sternites). Moreover, the upper side of the elytra was able to keep an air film up to flow velocities of 5 m/s. Obviously the complex surface structure with tiny dense microtrichia and two types of larger specially shaped setae is relevant for this outstanding ability. Besides high air film persistence, the observation of a considerable fluid velocity directly at the air–water interface indicates the ability to reduce friction significantly. The combination of these two abilities makes these hierarchically structured surfaces extremely interesting as a biomimetic model for low friction fluid transport or drag reduction on ship hulls.

## Introduction

Superhydrophobic surfaces are of great economic interest because of their amazing properties. In nature they occur in many species of animals and plants [1–2]. These surfaces combine a special topography at the micro- and nanoscale with a superhydrophobic surface chemistry [3–4]. Transferred to technical surfaces, superhydrophobic surfaces have successfully entered the markets of the world [5–6]. The effective self-cleaning mechanism of the Lotus flower *Nelumbo nucifera* is especially well known [3]. Granting of a patent in 1998 [7], followed by the introduction of the trade mark Lotus-Effect^®^ was the start of the realisation of biomimetic self-cleaning surfaces. Another highly interesting property of superhydrophobic surfaces, which did not receive so much attention in the past, is the ability to retain an air film while submerged under water. This air film cover can reduce drag of solid bodies sliding through water [8–9]. Therefore, air retaining surfaces are of great economic and ecological interest for low friction fluid transport and friction reduction on ship hulls [10–12]. On some technical superhydrophobic surfaces extremely high drag reduction of up to 50% was measured, but on these surfaces the air film lasted only a short time [13–15].

**Figure 1:**
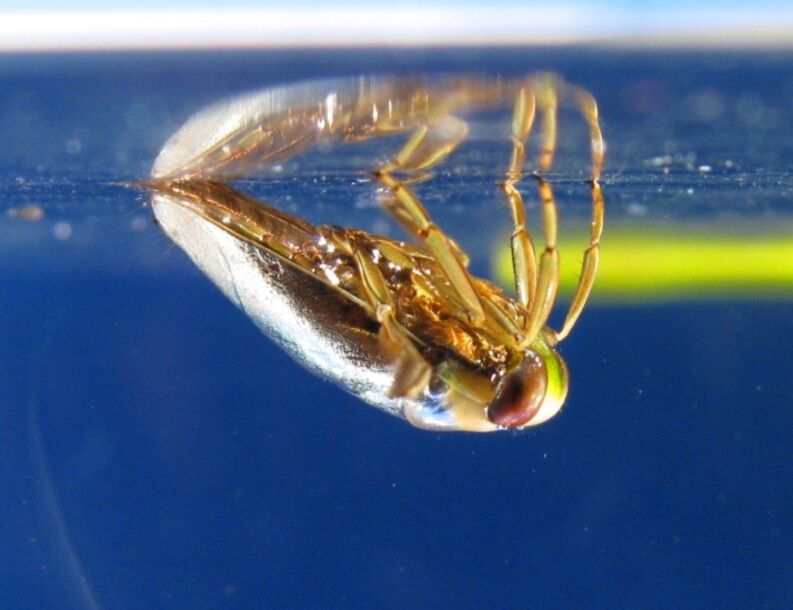
Lateral view on the water bug *Notonecta glauca*.

Biological air retaining superhydrophobic surfaces in the past were primarily examined in the context of their respiratory function [16–21], while some recent publications deal more with their functional morphology and their suitability for technical application [11,22–24]. For biomimetic applications of air retaining surfaces for low friction fluid transport and drag reduction on ship hulls, the durability of the air film is most important. While on many superhydrophobic surfaces the air film usually lasts no longer than a few days, some semi-aquatic plants and insects are able to hold an air film for a longer time period [19,24–25]. Very high air film persistence was observed on the water bug *Notonecta glauca* under hydrostatic conditions on their elytra [24]. Based on this comparative study about the correlation of surface morphology and air film persistence on different semi-aquatic insects, we chose *Notonecta glauca* as the model organism for further investigations on air film persistence and drag reduction. The backswimmer *Notonecta glauca* is surrounded by a thin film of air covering most body parts and causing a silvery sheen (Figure 1).

*Notonecta* spp. can dive and swim quickly through water, but most of the time it supports itself from underneath against the water surface with both pairs of fore legs and the tip of the abdomen [26]. The surface of the elytra is covered by a hierarchical structure of larger setae and very small microtrichia. Balmert et al. hypothesized that the dense microtrichia cover is relevant for the high air film persistence of the elytra measured under hydrostatic conditions [24]. In the present study we will prove this assumption by comparing the different structures on the body parts of *Notonecta*. Moreover, we measure the persistence of the air film of the elytra under hydrodynamic conditions and its effect on friction drag. By carrying out scanning electron microscopy, particle image velocimetry and air film persistence tests, we will answer the following questions: (1) Do the air retaining surface structures vary on the different body parts? (2) Is there a correlation between surface structure and air film persistence? (3) How is it possible to hold the air film under hydrodynamic conditions? (4) How much is the friction on the elytron surface reduced?

## Results and Discussion

### Characteristics of air retaining surfaces

**Figure 2:**
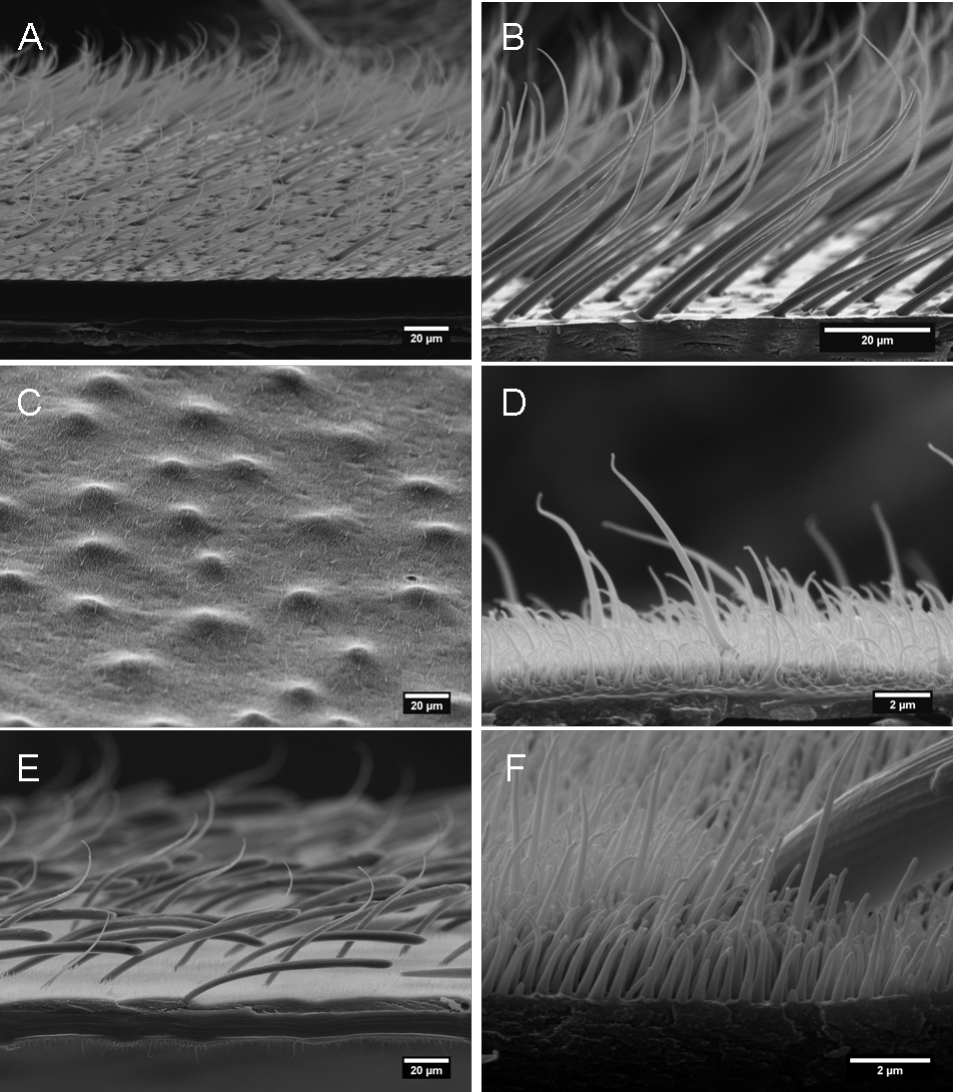
Selected air retaining body parts of *Notonecta glauca*: A,B) setae on the abdominal sternites; C,D) underside of the elytra with a dense cover of microtrichia; E,F) upper side of elytra with a hierarchical double structure of microtrichia and setae. Two different types of setae occur. In all pictures the caudal direction of the insect is on the right side.

*Notonecta glauca* is covered with hairy structures over almost all its body with exception of head, pronotum and legs. The body parts show a large variety of surface structures, but in general two types of surface protuberances occur: Large and sparse setae as well as small and dense microtrichia. Setae have a socket originating from an adjacent cell and are classified as true hairs, while microtrichia originating from one cell and are, by definition, not really hairs [27–28]. Three different body parts of *Notonecta glauca* with different surface structures were selected for further investigations; a pure setae structure, a pure microtrichia structure and a hierarchical structure with setae and microtrichia (Figure 2). On the abdominal sternites a pure setae structure can be observed. About 2,300 setae per mm^2^ cover the surface up to a height of approximately 30 µm. The basis of these setae points in the caudal direction while the tips are bent in the distal direction (Figure 2B). These surfaces are inside of the area of the air pockets on the abdomen.

In contrast, the underside of the elytra is covered only with microtrichia. These tiny protuberances of an average height of 1.2 µm show a large variation in height (Figure 2D) and reach a density of approximately 5.8 × 10^6^ mm^−2^. The underside of the elytra is only able to hold a very small volume of air due to their minor height. The little air film, however, might primarily help to keep the wings dry.

The upper side of the elytra is hierarchically structured by larger setae and tiny microtrichia (Figure 2E and Figure 2F). The microtrichia cover shows a similar density (6.0 × 10^6^ mm^−2^ on average) as the underside of the elytra, but the height is somewhat larger (2.3 µm). On these surfaces two different types of setae occur. The first seta-type (ST 1) is tapered and bent while the tips point more or less in the anterio–distal direction. In contrast, the second seta-type (ST 2) is clubbed and points in the posterior direction. The combined density of both setae-types is approximately 250 mm^−2^. The air film on the upper side of the elytra might help to keep the wings dry and save air for respiration, but friction reduction might also be an additional advantage for the backswimmer while hunting. An overview of the structural parameters of the investigated surfaces is given in Table 1.

**Table 1:** Structural parameters of the investigated body parts of *Notonecta glauca* (mean ± 95% confidence interval, N = 6).

	sternites	elytra (underside)	elytra (upper side)		
			ST 1 (pins)	ST 2 (clubs)	total

**setae**					
height [µm]	30 ± 3	—	60 ± 15	17 ± 5	39 ± 7
length [µm]	51 ± 6	—	85 ± 4	95 ± 5	90 ± 4
density [mm^−2^]	2,332 ± 359	—	90 ± 14	163 ± 30	253 ± 18
diameter [µm]	2.3 ± 0.2	—	3.1 ± 0.2	3.4 ± 0.2	3.3 ± 0.1

**microtrichia**					
height [µm]	—	1.2 ± 0,2	—	—	2.3 ± 0,8
density [mm^−2^]	—	(5.8 ± 0.3) × 10^6^	—	—	(5.9 ± 0.2) × 10^6^
diameter [µm]	—	0.32 ± 0.05	—	—	0.38 ± 0.05

All investigated surfaces are more or less superhydrophobic (Table 2). Contact angles ranged between 154° and 158°. The tilting angles were less than 5° on the sternites (not measurable) and the underside of the elytra. On the upper side of the elytra the tilting angle was higher (15°), which might be explained by the distant shape of the first seta-type (ST 1). However, in all cases a Cassie–Baxter regime [29] can be assumed so that the applied drop rests on an air layer in between the cover of surface protuberances. These hydrophobic structures enable the animal to trap an air film between the bottom surface and the tips of the surface protuberances.

**Table 2:** Contact and tilting angle of the investigated body parts of *Notonecta glauca* (mean ± 95% confidence interval, N = 10).

body part	contact angle [°]	tilting angle [°]

elytra (upper side)	154.2 ± 1.6	15.0 ± 4.3
elytra (underside)	155.3 ± 4.9	<5
sternites	157.5 ± 32	<5

### Air film persistence under hydrostatic conditions

Submerged in an aquarium with freshwater, the three described surface structures showed large differences in air film persistence. Experiments were performed with natural surfaces as well as with an additional superhydrophobic coating ("Antispread") on the original surfaces in order to exclude an influence of a possibly different surface chemistry. The results show similar air film persistence on untreated and treated surfaces (Table 3). Consequently, the surface structure can be identified as the relevant feature.

**Table 3:** Air film persistence of the submerged body parts of *Notonecta glauca* (N = 10).

body part	untreated sur- face [d]	surfaces treated with "Antispread" [d]

elytra (upper side)	>130	>130
elytra (underside)	>130	>130
sternites	<2	<1

**Figure 3:**
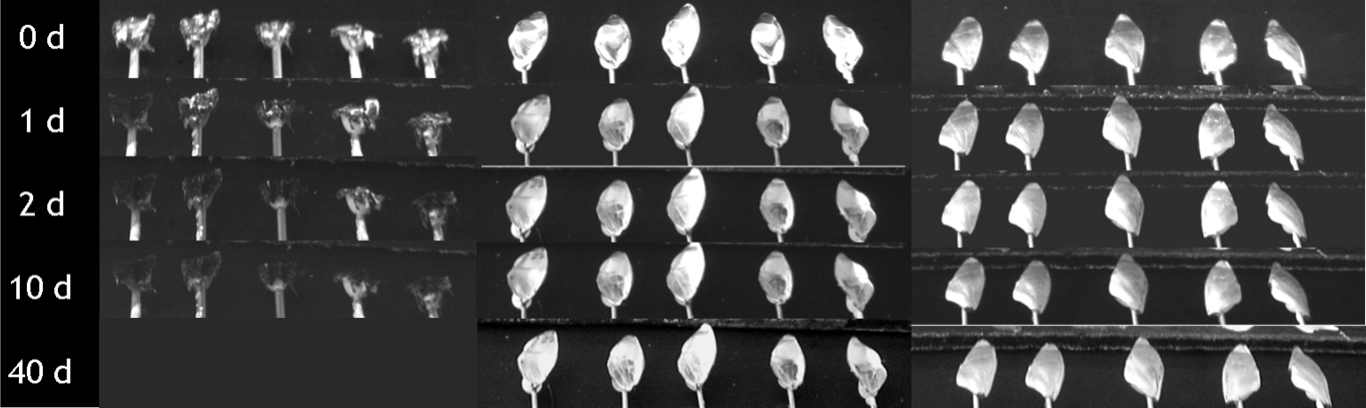
Submerged body parts of *Notonecta glauca* in the course of time. All surfaces were treated with a hydrophobic coating. Left side: sternites; middle: underside of elytra; right side: upper side of elytra.

**Figure 4:**
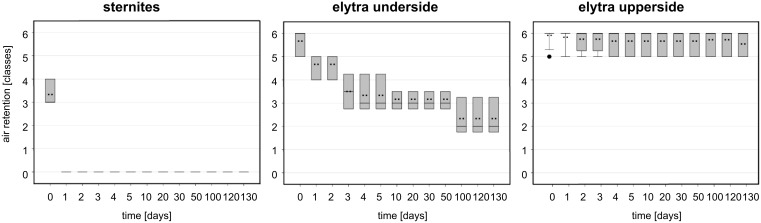
Air retention [classes] of the submerged surfaces of *Notonecta glauca* vs time. All surfaces were treated with a hydrophobic coating. Air retention classes define the air retaining portion (X) of the surface, with 0: X = 0%; 1: 0% < X < 20%; 2: 20% ≤ X < 40%; 3: 40% ≤ X < 60%; 4: 60% ≤ X < 80%; 5: 80% ≤ X < 100%; 6: X = 100%.

The air covering the surfaces can be identified by its silvery sheen [30–32]. On both sides of the elytra the air film remained for months, whereas it vanished after 1–2 days on the pure setae structures of the sternites (Table 3, Figure 2). On the upper and underside of the elytra air was still present after 130 days. This time span is much longer than that reported for *Salvinia* species, which are able to hold air for only weeks. Nevertheless, the quality of the air film was not the same on both surfaces of the elytra. After 130 days, the upper side of the elytra was still covered with air, almost totally. In contrast, the underside of the elytra was no longer completely covered with air (Figure 3 and Figure 4). In the latter, little gaps in the air film cover were identified after a few days or a week, which increased with time.

The different air retaining abilities of the investigated body parts of the backswimmer indicate an optimization for different functions. The short air film persistence of the pure setae structure (about 2 d) corresponds with the air retaining properties described for more or less similar structures on the abdominal sternites of other water bugs (*Ilyocoris*, *Corixa*) [24]. These surfaces might be optimized to hold additional air for respiration. The volume of the air might be more relevant than long term stability of the air film because the air store in this area is frequently renewed.

The underside of the elytra with a dense but pure microtrichia cover showed high air film persistence. This observation underlines the high relevance of the dense cover of surface protuberances previously assumed by Crisp in 1949 and proved by Balmert [24,33]. The wetting of the microtrichia cover will require a significant amount of energy due to the density of the structures. A dense cover of surface protuberances leads to a smaller radius of curvature of the air–water interface between the structures: More energy is necessary to displace an interface with a smaller radius [19,24]. Furthermore, the variable height of microtrichia leads to an increased density of microtrichia with decreasing distance to the body surface. Also the decreased air–water interface itself leads to a higher resistance against water pressure according to the model of Crisp and Thorpe [17]. The tiny, but stable, air film on the underside of the elytra seems to be more relevant to keep the wings dry rather than playing a role in the storage of air required for respiration.

Nevertheless, our results show that the quality of the air film was not the same as for the upper side of the elytra. Therefore, it can be hypothesized that the presence of setae in addition to the microtrichia cover not only contributes to the storage of a higher volume of air, but also stabilizes the air film itself by a kind of two barrier system. Also the reduced height of the microtrichia on the underside (1.2 µm) in comparison to the upper side of the elytra (2.3 µm) might contribute to the reduced air film persistence. In contrast to the underside of elytra in the living animal, the upper side stays in direct contact with the water. It therefore may have developed further adaptations to stabilize the air film. Obviously, the hierarchical double structure of the upper side of the elytra with a dense microtrichia cover and two different kinds of setae is most advantageous for long term air film persistence. Both types of setae are more or less tilted in the posterior direction at their bases. Therefore, a directional choice is forced when the setae are bent and a more stable air film can be expected according to Blow and Yeomans in comparison to setae bent in different directions [34].

### Air film persistence under hydrodynamic conditions

**Figure 5:**
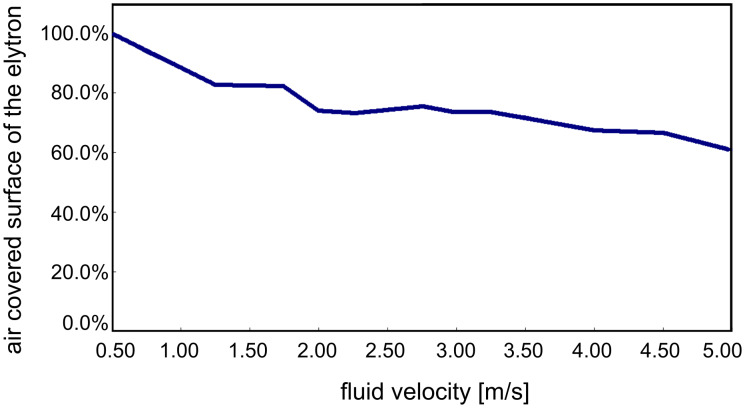
Air covered surface on the upper side of the elytron at increasing inflow velocity.

Due to their outstanding properties in air film persistence under hydrostatic conditions, the upper side of the elytra was chosen for hydrodynamic experiments. Impressively the upper side of the elytron was able to hold an air film up to a fluid velocity of 5 m/s (Figure 5).

The experiment started at low flow velocities of 0.5 m/s where no wetting was visible. At increasing flow velocities the fraction of the surface on the elytron covered with air decreased slightly (80% at 1.5 m/s). It is remarkable that at inflow velocities between 1.5 m/s and 3.5 m/s the fraction of air covered surface is reduced only marginally by 10% (70% at 3.5 m/s). Even at high flow velocities of 5.0 m/s, 61% of the surface initially covered with air is still intact. In comparison, the water fern *Salvinia molesta,* which is another model organism for air retention, had about 60% of the initial area covered with air at a flow velocity of 2.25 m/s [35]. Obviously, the surface structure of the upper side of the elytra of the backswimmer is optimally adapted to hold an air film under hydrodynamic conditions. Beneath the dense microtrichia cover the special shape of the two different seta-types seems to contribute to this remarkable property. The angular positioning of the setae in the direction of flow leads to an increased contact area between setae and water when the setae are bent by increased pressure due to pressure fluctuation. Therefore, a similar mechanism as described for the eggbeater shaped structures of *Salvinia molesta* [12] occurs. The penetration of the water requires energy for creating the larger contact area between the hydrophobic setae and water. The bending of the setae might also enable a flexible adaptation of the air water interface to the oscillation of the water flow. On the elytron surface, moreover, the microtrichia cover is present as an additional effective backup-system, if water is pressed close to the body surface between the setae.

### Velocity field above the elytron surface under hydrodynamic conditions

**Figure 6:**
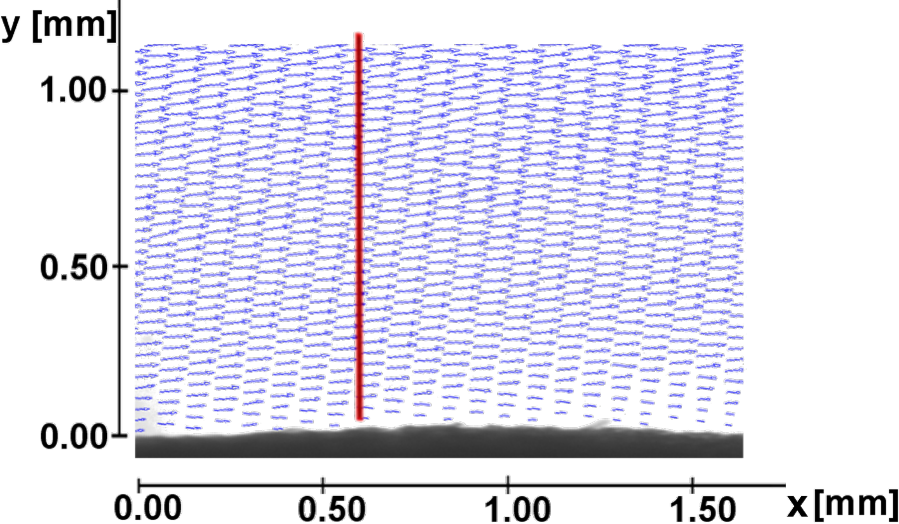
Averaged velocity field over the elytron surface (upper side).

**Figure 7:**
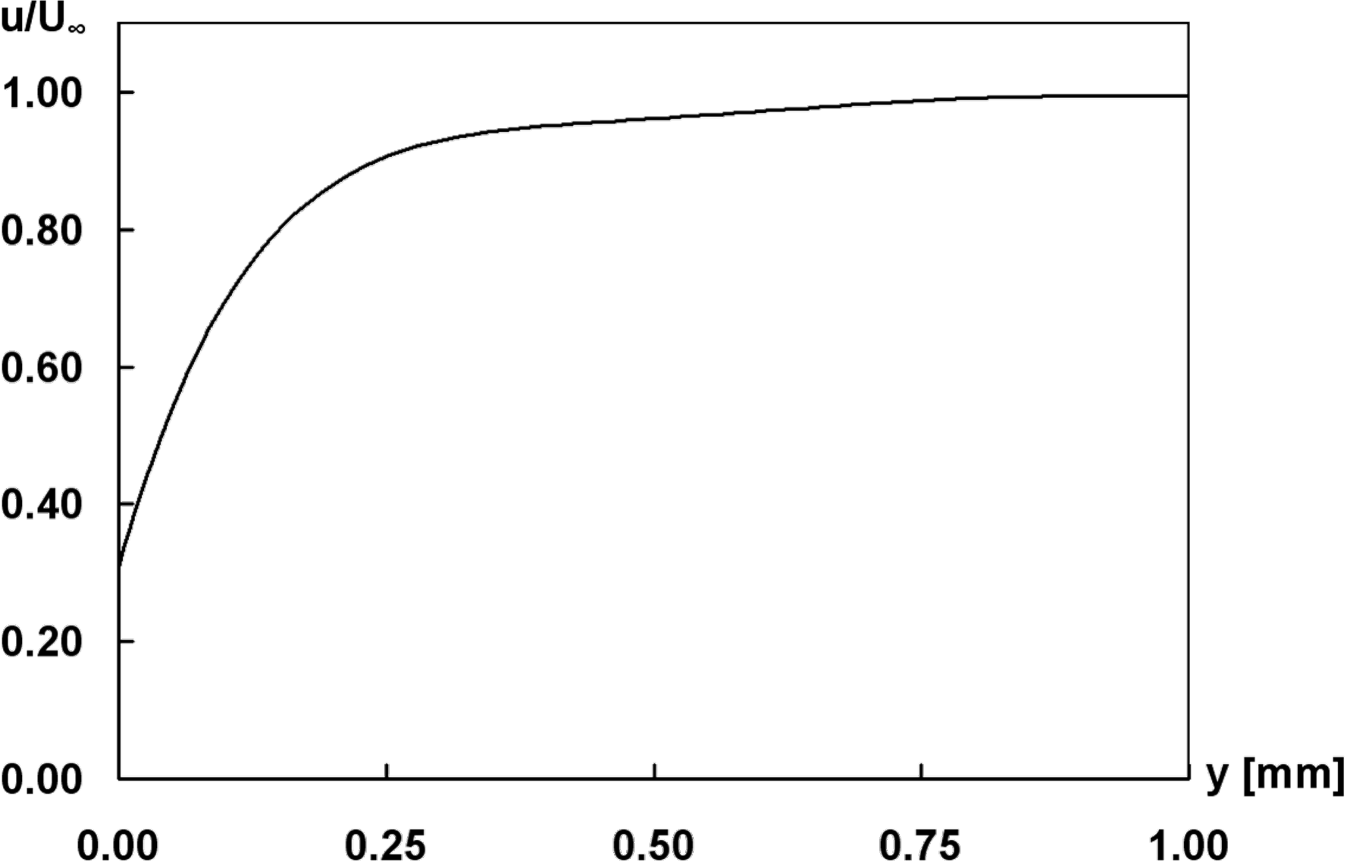
Velocity component u parallel to the elytron surface recorded along path in Figure 6.

Next to the persistence of the air film at the surface, the quantification of the velocity field and the friction reduction at the water–air interface are most important. The velocity field above the elytron surface was investigated by means of particle image velocimetry (PIV), which is a contactless method to measure the velocity field in fluids by analysing the particle movement. The measurement of the flow on the elytron surface were performed at a flow velocity of 0.5 m/s parallel to the surface. To represent the mean flow field above the elytron surface, the average of the velocity vector measurements using PIV is presented in Figure 6. For a better survey only every second velocity vector in the vertical and horizontal directions is plotted. Along the red line velocity vectors were selected to assemble a profile perpendicular to the surface. The resulting profile is depicted in Figure 7, the values being normalized with the mean velocity of the oncoming flow. The profile shows that the flow velocity in the vicinity of the surface differs significantly from zero due to the air retained on the surface. Since wall bonding is not possible at the water–air interface, slippage can be observed.

The velocity measurements indicate that the fluid moves over the interface at nearly one third of the inflow velocity. Hence, it can be assumed, that a considerable friction reduction is present. Next to the effect of the air film itself on friction reduction, we assume that the special shape of the setae on the upper side of the elytra might contribute to this property.

## Conclusion

Our investigations showed outstanding air film persistence under hydrostatic and hydrodynamic conditions of the upper side of the elytra of *Notonecta glauca*. Moreover, a considerable reduction of the friction on the elytron surface was measured. The combination of these two abilities makes these hierarchically structured surfaces extremely interesting for biomimetic applications such as low friction fluid transport or drag reduction on ship hulls.

## Experimental

***Animals.*** Freshly killed insects were dissected and the air retaining surfaces were processed immediately after taxidermy.

***Scanning electron microscopy.*** Isolated body parts were glued on insect pins, sputter-coated with 20 nm gold on the upper and underside (Balzers Union SCD 040 Sputter-Coater, Baltec AG, Liechtenstein) and screwed to a custom made holder [25,36], which allowed the examination of all sides. The specimens were examined with a Cambridge Stereoscan 200 scanning electron microscope (SEM) (Cambridge Instruments Ltd., Cambridge, UK) using a tungsten cathode and accelerating voltages between 5 and 15 kV. To determine structural surface parameters, SEM images of 10 areas of each of the 6 investigated individuals were analysed with the digital image processing software ImageJ.

***Contact angle (CA) measurements.*** To characterise the wetting behaviour of the biological surfaces, contact (CA) and tilting angle (TA) measurements were performed with a DataPhysics Contact Angle System OCA, TBU 90 E (DataPhysics GmbH, Filderstadt, Germany), which was operated by the software SCR 20 (DataPhysics). CA measurements were performed with the “needle-in-drop” method by application of 5 µL droplets of pure water. CA and TA were measured for ten drops to each of ten individuals.

***Measurement of air film persistence under hydrostatic conditions.*** For this experiment, three different body parts of *Notonecta glauca* with different surface structures were selected. Ten samples of each surface were fixed on insect pins glued onto thin plates of polyvinyl chloride by a water resistant two-component adhesive (Pattex Powerkleber Kraft Mix Extrem Schnell, Henkel AG & Co. KGaA, Düsseldorf, Germany). Specimens were submerged in an aquarium filled with pure water to a depth of 15 cm at room temperature. Air film persistence was detected optically by light reflection at the air–water interface. Digital images of the submerged specimens were recorded using a Canon Power Shot SX110IS digital camera (Canon Inc., Japan).

To compare the air film persistence of the three investigated surfaces with regard to the different surface structures, we covered them with a uniform hydrophobic surface coating. For that purpose the surfaces were dipped in Antispread (F2*/*200 Fluorcarbon 60, Horb-Ahldorf, Germany) for 10 s which forms approximately 40 nm thin layers on the substrate surfaces (product information). After drying for 30 minutes, the hydrophobized specimens were treated like the native surfaces as described above.

***Measurement of the velocity field above the surface and air film persistence under hydrodynamic conditions.*** Experiments under hydrodynamic conditions were performed for the upper side of the elytra by particle image velocimetry. Neutrally buoyant particles were added to the flow and then digital images were recorded in selected planes [37]. For macroscopic setups, the location of the plane was defined by illuminating the particles with a thin laser-light sheet whereas for microscopic experiments the plane was selected by the depth of focus in a completely illuminated volume [38].

The experiment was performed in a closed circuit water channel with a cross-section of 15 × 30 mm^2^. A single elytron of *Notonecta* was fixed to an exchangeable retainer which was placed inside the channel using a streamlined base plate on a wall mounted post (Plastik Fermit weiß, Fermit). For the observation of the flow, a microscope with a Leica lens (planapo 2.0) was used resulting in an observation area orthogonal to the elytron surface of 2.6 × 1.4 mm^2^. Within the focal plane of the lens, the particle images could then be observed. Double-frame image pairs were generated by a HiSense PIV camera (Dantec Dynamics, Copenhagen, Denmark) while the flow-field was completely illuminated by a pulsed 5 W LED. The in-plane velocity vector components were then calculated using an average correlation scheme with an interrogation area size of 64 × 32 pixels and an overlap of 50%. Finally, the velocity profiles of the flow along a path perpendicular to the air retaining surface were evaluated.

In addition to the analysis of the velocity field the persistence of the air film under dynamic conditions was determined. To quantify the air film coverage on the elytron, images of the surface of the elytron were taken with a digital SLR camera (Nikon D80, Japan) at different flow velocities in the channel. Additional illumination was achieved by a Cold-light source KL 1500 LCD (Zeiss, Germany). The dimension of the air film on the surface was subsequently derived from the images using the area of total light reflection on the elytron.
